# Report of a Case of Inflammation and Ulceration of the Gums and Exfoliation of the Alveolar Processes

**Published:** 1850-07

**Authors:** Alfred A. Blandy


### ARTICLE VII.

*Report of a Case of Inflammation and Ulceration of the Gums and Exfoliation of the Alveolar Processes.* By ALFRED A. BLANDY, M. D., D. D. S.

Mr. D——, a hard-working man, of sanguino-bilious temperament, forty-five years of age, of usually good habits, possessing a strong constitution, without any previous illness, being compelled to work in water in

February, 1849, contracted a severe cold, followed by pain in his teeth. With little attention afterwards, he continued his employment for three weeks. By this time the pain had extended to all his teeth and mouth generally. This continuing to increase, he sought medical advice; the gums were much swollen and painful. An opiate and wash for his mouth was prescribed. The next day found him much worse; face disfigured from tumefaction; pulse strong, full and frequent: mind flighty,—the inflammation and swelling had extended to the palate and fauces, preventing mastication and impeding deglutition. Sixteen ounces of blood were taken from the arm, and calomel administered, followed by some other strong cathartic; next day this was repeated without any beneficial change; the gums were lanced, discharging a large quantity of purulent matter.

This state of things had now continued for about two weeks, the mouth remaining in nearly the same condition. The whole system became implicated in the disturbance, accompanied by acute hepatitis, with painful dysuria.

These symptoms continued for about fourteen days, giving way at last to treatment, when the patient, by the advice of his physician, called upon me. The gums and teeth at this time presented a most dreadful appearance. The former were swollen, of a livid color, assuming a sloughy aspect; the latter were all loose, and the alveoli in a necrosed and partially exfoliated condition; the patient experienced great pain whenever he attempted to close his mouth; said his jaws were dead, and felt as if they were coming away. Some of the worst of his teeth were removed, and gums freely scarified, recommending a generous diet, with occasional use of mild saline purgatives, and astringent gargles. In a few days several pieces of bone came away, together with the second left superior bicuspis and first molar. The sockets of these teeth soon exfoliated. The teeth and alveolar processes from this point, around the whole arch to the second right bicuspis, being in a loose, vacillating condition, I separated the imperfect attachment that existed between them and the gums, and removed the entire alveolar border. The other treatment as above described was continued, followed by an improvement of the general health. Several pieces of bone were, however, afterwards thrown off.

The gums still remaining spongy and manifesting but little disposition to heal, were touched with caustic. This soon induced healthy granulations, but the palatine mucous membrane still remained loose and sensitive, but with the restoration of the general health, these symptoms gradually disappeared, leaving the mouth, however, much disfigured, and articulation greatly impaired. At the expiration of eight months, the lost parts were replaced with an artificial substitute, consisting of a raised or box plate, upon which porcelain teeth were mounted, secured in the mouth with strong clasps, adapted on each side to a molar tooth. This completely restored the contour of the face, enabling him to articulate distinctly, and very greatly assisted in mastication.*

*The exfoliated portions of the alveolar border and teeth which they sustained, have been placed in our hands, by the writer of the above report.—
*Balto. Ed.*

